# Animal Models for the Study of the Relationships between Diet and Obesity: A Focus on Dietary Protein and Estrogen Deficiency

**DOI:** 10.3389/fnut.2017.00005

**Published:** 2017-03-20

**Authors:** Tristan Chalvon-Demersay, François Blachier, Daniel Tomé, Anne Blais

**Affiliations:** ^1^UMR Physiologie de la Nutrition et du Comportement Alimentaire, AgroParisTech, INRA, Université Paris-Saclay, Paris, France

**Keywords:** animal models, obesity, body composition, dietary protein, food intake, energy expenditure, estrogen deficiency

## Abstract

Obesity is an increasing major public health concern asking for dietary strategies to limit weight gain and associated comorbidities. In this review, we present animal models, particularly rats and mice, which have been extensively used by scientists to understand the consequences of diet quality on weight gain and health. Notably, modulation of dietary protein quantity and/or quality has been shown to exert huge effects on body composition homeostasis through the modulation of food intake, energy expenditure, and metabolic pathways. Interestingly, the perinatal window appears to represent a critical period during which the protein intake of the dam can impact the offspring’s weight gain and feeding behavior. Animal models are also widely used to understand the processes and mechanisms that contribute to obesity at different physiological and pathophysiological stages. An interesting example of such aspect is the situation of decreased estrogen level occurring at menopause, which is linked to weight gain and decreased energy expenditure. To study metabolic disorders associated with such situation, estrogen withdrawal in ovariectomized animal models to mimic menopause are frequently used. According to many studies, clear species-specific differences exist between rats and mice that need to be taken into account when results are extrapolated to humans.

## Introduction

Obesity is a worldwide epidemic affecting over 400 million adults with serious comorbidities (1). Obesity develops when energy consumption exceeds energy expenditure and is defined as the accumulation of excess body fat to the extent that its results in health complications and reduces life expectancy (2). As obesity prevalence is rising, the quest to find new treatments to diminish its negative consequences is also increasing. Experimental research needs to determine the mechanisms by which obesity increase the risk of diseases. To investigate the interactions between the components of the diet and the biological processes, epidemiological, experimental, and clinical studies are necessary.

Regarding experimental studies, animal models are essential for *in vivo* and *ex vivo* experimental design. Nutrient and non-nutrient components of food interact with many metabolic pathways at different levels including gene expression regulation. Experimental models, from cells to organoids and animals, are also essential to elucidate mechanisms by which food components can modulate metabolic pathways. To be able to translate, at least partly, the information obtained from an animal model to humans, the choice of the appropriate animal model is a crucial step to avoid as much as possible misinterpretations.

Dietary interventions studies in animals are thus essential to understand the biological roles of specific nutrients before validation in human. In the last century, rats were the most used in biochemical research, but in the last two decades, its popularity decayed due to the limitation to perform reverse genetics in rats*. Mus musculus* is probably the most popular model used to identify the mechanisms of food intake and energy regulation. Even if some extrapolation from mice to humans is hazardous, the mice model has helped us to develop some therapies for obesity, metabolic syndrome, and insulin resistance (3). If mouse models obviously do not mimic all aspects of human diseases, they are, however, the most commonly used models. No other animal model offers such large possibilities of phenotyping in response to metabolic, genetic, and behavioral manipulations. Depending on the target, the most widely used mouse models are (i) spontaneously occurring obese mouse strains that are well characterized, (ii) high-fat diet that rapidly induce weight gain in mice, and (iii) transgenic or gene knockouts mice to determine the influence of a given gene in the development of obesity.

Animal models have thus been used extensively by the scientific community to understand the role of diet quality on health. A better understanding of the relation between diet quality, and also physical activity and progression of chronic disease, such as obesity as presented in this review article, is increasingly important with regard to the increase in the number of obese individuals worldwide.

This review will focus specifically on two typical situations in the rodent models: (i) the impact of protein quality/quantity and (ii) the impact of estrogen deficiency on body weight and composition. A short section on the pig model will conclude this article to summarize the advantages and limitation of this model versus the rodent models, in general terms and in terms of studies on obesity.

## Protein Quantity/Quality

Dietary intervention studies in animal models are essential to understand the biological roles of specific nutrients before validation in humans.

### Dietary Protein Intake

Protein is an essential dietary component in which recommended level is defined as the minimum intake required to maintain nitrogen balance; and as the amount of protein sufficient to prevent the catabolism of body protein stores. The recommended daily minimum intake of protein and amino acids (AAs) in adults is 0.8 g/kg of body weight (4). However, recent studies using stable isotope suggest that current dietary protein recommendation may not be sufficient to promote optimal muscle physiology in all populations (5). Epidemiological studies support the notion that especially in the older population, a greater protein intake, up to 19% of the energy, better preserves lean body mass (LBM) (6). In industrialized countries, the main sources of protein are milk, eggs, and meat. The nutritional value of protein is influenced by several factors, especially the AA composition, protein digestibility, protein digestion kinetics, and the ability to transfer AA for protein synthesis. Diets based on either animal or vegetable products supply proteins of different quality in different quantities. Plant proteins are often lower in some specific indispensable AAs when compared to animal proteins. For instance, soy protein is reported as a “complete” protein, but its overall indispensable AA content is lower than the one measured in milk proteins (7). Thus, protein quality, which is defined as the capacity of dietary protein sources to satisfy the metabolic needs for protein, and as the content in essential AAs, is important when considering protein requirements. Correlations between protein nutrition and human health are becoming a highlighted research topic.

### Low-Protein (LP) and High-Protein (HP) Diets

Studies have suggested that when rats are placed in food choice position, they regulate their protein intake, so that it corresponds to their nutritional needs (8). Consistent with these results, experiments have shown an increase in food intake when the diet protein content is decreased at the expense of carbohydrates (9). The “protein leverage hypothesis” issued by Simpson and Raubenheimer proposes that paradoxically, proteins, which only represent between 10 and 15% of the average energy intake in adults represent the key factor in body weight and composition regulation (10). These authors have observed that the ratio between the protein and other nutrients (carbohydrates and lipids) has dropped in the last years. Thus, people, according to their hypothesis, tend to consume more dietary proteins to cover their protein needs. This excessive consumption of HP and LP density food, may partly explain the weight gain and obesity measured in these individuals. This observation is in line with numerous animals studies showing that substitution of carbohydrates by proteins in HP diet reduce adiposity and food intake (11, 12), while LP diets are associated with an increase in food intake and fat mass (13–15).

Since LP and HP diets are commonly consumed, it is particularly interesting to study consequences of those diets on human health. However, it should be underlined that the average amount of dietary protein consumed is generally above the recommended dietary intake in Western countries. For instance, in France, the average dietary consumption is 1.7-fold the recommended dietary intake (16). The consumption of HP diets, which can lead to the consumption of dietary protein up to four times the recommended dietary protein intake, are frequently used by individuals who wish to decrease their body weight. Although body weight diminution in overweight and obese individuals is obviously associated with beneficial outcomes, some deleterious effects of HP diet have been suggested in several studies. Indeed, HP diets are contraindicated for individuals who are suffering or predisposed to kidney diseases (17). Regarding the intestinal physiology, in case of HP consumption, a part of dietary and endogenous proteins escapes full digestion in the small intestine and is transferred to the large intestine, where they are metabolized by the intestinal microbiota that produce, from AAs, various metabolites, some being beneficial, while most of them being deleterious when present in excess (18). An epidemiological analysis performed by Shoda et al. (19) in Japan showed a correlation between incidence of Crohn’s disease (an intestinal chronic inflammatory bowel disease) and increase intake of animal protein over a period of 20 years. A prospective cohort study carried out in women also reported a positive association between the level of dietary intake and risk of inflammatory bowel diseases (20). However, Spooren et al. have recently performed a systematic review of the epidemiological studies that have examined the links between protein intake and the risk of developing inflammatory bowel diseases and have reported that most studies performed found no significant association between these two parameters (21). When interpreting the results of these different studies, it is worth taking into consideration that, due to the high complexity of diet, it may appear difficult to collect robust dietary data. Then, it remains possible that the effects of the protein intake on the risk of developing inflammatory bowel diseases may have been biased in some studies by confounding factors.

Regarding the possible links between HP diet and the risk of colorectal cancer, the results obtained from epidemiological and experimental studies do not allow to reach any robust conclusion, the results obtained being rather heterogeneous (22). Observational studies have reported that HP diet is linked with higher mortality due to cardiovascular disease (23, 24). However, as discussed earlier, increased protein consumption is commonly associated with high intake of other alimentary compounds like meat that contains notably heme, *N*-nitroso compounds, and heterocyclic amines, which have been reported to exert negative effects on various health aspects when present in excess. Therefore, these confounding factors do not allow to determine clearly the role of protein *per se* on various health parameters.

Concerning LP diets, there is no clear definition for this type of diet. LP diets are often recommended for patients with anomalies of the AA metabolism including phenylketonuria and those with kidney or liver diseases (25, 26). Furthermore, in developing countries, children during fetal development, lactation, and after weaning are often fed with diets including high carbohydrate but LP level (27, 28). It is therefore important to determine the effects of LP diets on weight and body composition.

The consequences of HP and LP diets on body composition can be studied in animal models with no difference in energy content. For example, experimental HP diets usually contain less digestible carbohydrates but had exactly the same composition regarding lipids, undigestible carbohydrates, minerals, and vitamins (29). The use of animal models is suitable to reveal the underlying mechanisms.

### Dietary Protein Intake, Body Weight, and Composition

Numerous studies have reported that HP diets allow reduction of adiposity while maintaining LBM in animals (30–32). In rats, it has been shown that HP diets, in which 50% of the energy is provided by proteins, drastically reduced after 6 months the white adipose tissue compared to a normal protein diet (30). Consistent with these results, Pichon et al. found that increasing protein level in the diet reduced weight gain more strongly than the reduction of carbohydrates/lipids ratio (32). Moreover, this decrease in weight gain was associated with decreased adipocyte size (11).

It has been shown that protein restriction can replicate the effects of calorie restriction over a short period of 8 weeks in mice, with a decrease in circulating insulin, glucose tolerance, and weight gain (15). However, consumption of LP diet over longer periods in mice is generally associated with increased weight, adiposity, and intrahepatic fat (14, 15). On the contrary, growing rats fed for 15 days with a LP diet exhibited a lower body weight but a greater adiposity (13), enlightening the importance of the dietary intervention duration.

### Dietary Protein Level and Food Intake

Mellinkoff, according to his aminostatic theory (33), was the first to hypothesize that the fluctuations of plasma AA concentrations could act on the control of food intake. Noting that «a rise in the serum amino acid concentration appears to be accompanied by a waning of appetite», he hypothesized that when plasma AA concentrations reach a threshold, satiety occurs. Furthermore, it is well known since several years that the AA content in the cerebrospinal fluid reflects circulating AA levels, itself linked to the dietary protein composition (34). Some of these AAs can also serve as precursor of neuropeptides that are directly involved in food intake regulation. This is the case for tryptophan, which is a precursor for serotonin, this latter neurotransmitter repressing food intake (35). Similarly, histamine is synthesized from histidine, and high levels of histidine are thought to have a negative effect on food intake through histaminergic neurons activation (36). AA composition could therefore explain why some proteins have been reported to be more satiating than lipids and carbohydrates (37, 38).

Taken together, these observations support the concept that a HP diet decreases food intake, while a LP diet increases it. On the contrary, very LP diet generates an aversive phenomenon (39) that allows the individuals to direct its choice toward balanced food to maintain essential AAs homeostasis (40).

The effects of HP diets on food intake are especially observed during the first days after the introduction of the diet. Once the animals are accustomed to the HP diet, they tend to return to a food intake similar to the one observed in animals fed a control diet.

The effect of both HP and LP diet on food intake is mediated by gastrointestinal peptides. Thus, Batterham and colleagues observed an increase in the plasma levels of the anorectic peptide PYY in mice following ingestion of an HP diet (41). In the same study, they have shown that mice deleted for the PYY gene no longer exhibit a decrease in food intake under a HP diet. In humans, studies have reported that HP diet is also associated with increases in the concentrations of glucagon-like peptide-1 (GLP-1), cholecystokinin (CCK), and a decrease in ghrelin concentration (42). Interestingly, LP diets are associated with small changes in CCK or ghrelin levels relative to control diets. Morrison and Laeger hypothesized that this blunted response may contribute to the hyperphagia observed in case of LP diet consumption (42).

The effect of LP diet on food intake is also mediated through FGF21 secretion. Indeed, the increase in food intake induced under a LP diet is suppressed in FGF21-KO mice (43).

Moreover, the supplementation of some AAs (histidine, phenylalanine, tryptophan, alanine, glutamine, and arginine) can partially mimic the satiating effect of HP diet on food intake and/or gastrointestinal peptides secretion (44–51). For example, oral glutamine or arginine increases the secretion of GLP-1 and improves glucose tolerance in rodents (50, 51). Branched AAs, particularly leucine, can reproduce the effects of a HP diet. Leucine supplementation in the diet or in the drinking water reduces food intake in rats and mice (46, 52). Furthermore, leucine icv injection, but not tryptophan, threonine, methionine, lysine, and serine, reduces food intake and body weight (43, 53), indicating that at least part of the anorectic signal induced by leucine is generated centrally. Leucine and HP diet exert their effect *via* an increase of mTOR activity and a decrease of AMPK activity in the hypothalamus, which leads to an increase in the anorectic pro-opiomelanocortin and a decrease of the orexigenic NPY and AgRP in the arcuate nucleus of the hypothalamus (53–55).

### Dietary Protein and Reorientation of Metabolic Pathways

Consumption of a LP diet, providing only 5–6% of the energy as protein, increases food intake, adiposity, and intrahepatic fat content compared to a control diet (14, 15). The increase of the hepatic lipogenesis is correlated to an increase in the SREBP-1c transcription factor and of glycerokinase activity by 30 and 50%, respectively (56). However, using diet in which 10% of the energy was provided by proteins, Henagan et al. found that consumption of a LP diet is associated with a decrease in liver lipogenesis, and in particular of the expression of stearoyl-coenzyme A desaturase (SCD-1), FAS, and SREBP-1c (57). The higher protein level used by Henegan may explain these conflicting results.

The triglyceride (TG) accumulation in adipocytes may also be related to a decrease in lipolysis. Indeed, Buzelle et al. showed that lipogenesis in adipocytes is usually lower under LP diet, and that these cells do not respond to the lipolytic action of noradrenaline (58). Thus, the disability to mobilize fat likely explains the accumulation of TG in white adipose tissue.

As the adiposity reduction related to HP diet is partially mediated by food intake reduction, it is necessary to use a pair-feeding group of animals to adjust the caloric intake of rats fed with a normo-protein diet with the one measured in rats fed with a HP diet (12). However, even if the rats fed with the standard diet are pair fed, they still exhibit a higher adiposity than the HP fed rats. These results support the view that the effects of HP diet involve the reduction of lipogenesis.

Fourteen days after the introduction of a HP diet, gene expression of FAS and ACC in the liver was suppressed (59). Moreover, the expression of FAS and SREBP-1c in the liver of rats fed for 8 weeks with a HP diet compared to rats fed a NP or NP pair-fed diet was also reduced (11).

Studies have shown that under a normo-protein diet, all AAs that are deaminated are oxidized rapidly. By contrast, under a HP diet, only half of the deaminated AAs are oxidized, resulting in the generation of a “carbon skeleton reserve” in the form of α-keto acids (60, 61). Furthermore, high plasmatic AA level increases both insulinemia and glucagonemia, which stimulates gluconeogenesis. Indeed, several AAs, including cationic AAs, are known stimulators of insulin secretion (62, 63). Moreover, Veldhorst and colleagues observed an increase in gluconeogenesis in healthy men fed a HP diet (64). In rats, the increase in dietary protein induces the expression of PEPCK in the fasted and fed rats and of glucose 6-phosphatase, only in the fasted state. These results suggest an increase in hepatic glucose synthesis (65). Ketogenesis is another metabolic pathway by which the carbon skeletons derived from the AA deamination can be managed. In fact, in humans and animals, an increase in circulating ketone body levels (especially β-hydroxybutyrate) was observed in response to HP diet ingestion (64). Finally, HP diets also allow a renewal of glycogen stores and an increase in the conversion of dietary AAs into glycogen (60, 66, 67).

### Dietary Protein and Energy Expenditure

Postprandial thermogenesis is defined as the increase in energy expenditure after a meal or after ingestion of a given nutrient. This parameter results from the energy cost corresponding to absorption, digestion, and metabolism of nutrients provided by the meal. In humans, it has been shown that postprandial thermogenesis is in the range of 15–30% of the ingested energy for protein, while for carbohydrates and lipids this value is, respectively, between 5 and 10% and 0 and 3% (68, 69). Mikkelsen et al. showed that when protein energy contribution in meal is increased from 11 to 29%, the energy expenditure is also increased of about 10% per day (70). The increase in energy expenditure associated with the consumption of protein may partly explain their satiating effect. Indeed, several authors have suggested that increased metabolism had an inhibitory effect on food intake (71, 72).

Recent studies report that in mice fed with LP or HP diets, the postprandial thermogenesis is increased compared to normo-protein diet (73). Similar results are reported for total energy expenditure (14, 74, 75). In line with these results, it has been shown that the basal temperature is increased by 1.1°C in animals fed a LP diet, and that administration of norepinephrine is more efficient to increase the basal temperature (+0.2°C) in rats fed a LP diet when compared to rats fed a control diet (76).

The effect of LP and HP diets on total energy expenditure is mediated notably by a modulation of genes encoding uncoupling proteins (UCP). UCP are proton carriers that uncouple their return into the mitochondrial matrix for ATP production, thus decreasing energy production efficiency. The energy from substrates oxidation is then dissipated as heat. Studies have reported an increase in UCP1 expression under LP (76) and HP diets (55). Another study found similar results in rats, showing that an increase in dietary protein intake is able to upregulate UCP2 expression in the liver. These changes that are associated with increased abundance in UCP are positively correlated to energy expenditure (75).

Both the effects of LP and HP diet on UCP expressions could be modulated by the restriction or supplementation of specific AAs, for instance, histidine supplementation increases the content of UCP1 in brown adipose tissue (44, 45).

On the other hand, Malloy et al. have shown that the energy expenditure in rats fed with a methionine-deficient diet was greater than that of rats fed *ad libitum* or in animals pair-feed to a control diet (77). Consistent with these findings, other studies have shown that methionine restriction was accompanied by an increase in energy expenditure, including thermogenesis and body temperature increase (78, 79).

**Figure 1:**
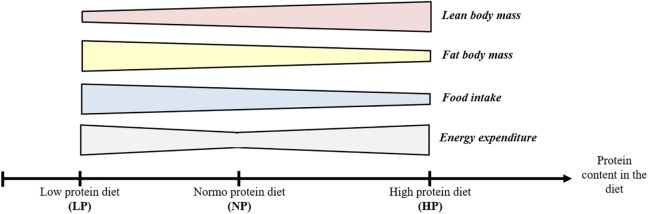
**Effects of low protein (LP) and high protein (HP) diet on body composition, food intake, and energy expenditure**.

Interestingly, the consumption of both LP and HP diets results in an increase in energy expenditure. The effects of the two types of diets are summarized in Figure 1 below.

### LP Diet and Protein Quality

Recent studies, using a new scoring system to qualify dietary protein quality, namely the digestible indispensable amino acid score (DIAAS), allowed a better comparison of protein quality. Indeed, this score is not truncated compared to usual systems used to evaluate protein quality like the nitrogen balance measurement. DIAAS classification is based on the relative digestible content of the essential AAs. This classification shows that dairy proteins have the highest quality (80). In accordance with this classification, a study in human volunteers has shown that milk proteins are more efficient to stimulate muscle protein synthesis than soy protein. The best effectiveness of milk protein for such an effect was correlated to the higher proportion of leucine (81).

Evaluation of the so-called ideal protein level to better maintain health is complex. Most animal studies designed to evaluate the impact of LP diet on health barely took into account protein quality, and this may explain discrepancy between different studies. A recent review of Le Couteur et al. (82) suggests that LP diets generate longer lifespans in *ad libitum*-fed mice. Interestingly, the same results were obtained using the *ad libitum* insect model, suggesting that these effects of LP diets apply to very different animal models. However, the protein leverage induced by LP diet, which increases food intake and fat deposition, is not always integrated. Restriction of particular AAs, such as methionine, has been shown to extend life duration in mice (83) and rat (84), and lower serum level of IGF-1, insulin, glucose, and thyroid hormone in serum (85).

A recent study using Balb/C mice under moderate protein restriction shown that protein quality is an important factor for biological effects. Ingestion of low quality protein that reduces IGF-1 serum level is related to decreased LBM and bone quality (86). This study included a group of control mice feed with a control soy-based normal protein diet including 20% of the total energy as soy protein (NP-SOY) and two other groups receiving LP diets. The first one was a soy-based protein restricted diet, with 6% of the total energy as soy protein (LP-SOY), while the second one was a casein-based protein restricted diet with 6% of the total energy as casein (LP-CAS). To avoid the protein leverage effect, a pair-feeding group corresponding to the LP groups was used. As all the diets were isocaloric, the pair feeding allowed to ensure that energy intake was similar in all the groups.

Over the duration of the experiment (60 days), total body weight of LP-SOY mice remained at the baseline value, while NP-SOY and LP-CAS mice gained weight. The difference in total body weight was related to a lower lean mass gain in LP-SOY when compared to LP-CAS and NP-SOY mice. Reduction of IGF-1 plasma level and bone quality related to reduce bone formation was observed in the LP-SOY group (Table 1) (86).

**Table 1:** **Effect of a soy- or casein-based protein restriction on body composition, bone quality, and bone turnover markers after 60 days**.

	Diets		
	NP-soy	Low-protein (LP)-CAS 6%	LP-soy 6%
Weight gain (g)	3.41 ± 0.48^a^	3.81 ± 0.36^a^	0.55 ± 0.40^b^
Lean mass gain (g)	2.05 ± 0.32^a^	1.57 ± 0.18^a^	−0.63 ± 0.26^b^
Uterus (mg)	81 ± 5^a^	52 ± 3^b^	24 ± 1^c^
Femur cortical thickness (mm)	0.232 ± 0.003^a^	0.226 ± 0.002^a^	0.205 ± 0.002^b^
Femur length (mm)	15.72 ± 0.12^a^	15.62 ± 0.13^a^	15.00 ± 0.11^a^
Femoral BMD change (delta%)	14.5 ± 0.5^a^	13.8 ± 0.9^a^	6.7 ± 1.5^b^
IGF-1	325 ± 30^a^	302 ± 18^a,b^	247 ± 15^b^
PINP (bone formation marker)	1.76 ± 0.15^a^	1.30 ± 0.13^a^	0.65 ± 0.06^b^

*Data are means ± SEM (*n* = 15). A one-way ANOVA followed by Bonferroni’s *post hoc* was performed. Means with different superscript letters (a, b, c) are significantly different (*p* < 0.05) according to a Bonferroni multiple comparison test (86)*.

The comparison of the effects of LP-SOY and LP-CAS diets on various parameters thus indicates that protein quality is of prime importance in the case of moderate protein restriction. The observed effects on body composition and blood plasma parameters could be partly related to a difference in AA profile, as casein is richer than soy primarily in methionine, and also in proline, serine, threonine, glutamine, valine, tyrosine, isoleucine, and leucine (87–89). Previous studies suggested that reduction of particular AAs in the diet can extend lifespan in mice and rats. However, this study shows that reduced IGF-1 level, which is correlated with reduction of bone formation and LBM including uterus weight, do have adverse consequences on health parameters (86). This latter study shows that LBM response to nutritional interventions, particularly dietary protein quality, is a good marker of the minimal dietary protein needed in this experimental model. This endpoint may be especially important for the aging population, because reduced protein intake which is often observed in the elderly, and which reduces LBM and bone mass are associated with fracture, reduction of life quality, and lifespan. However, data in humans also indicate that reduced protein intake may become an important component of anticancer and antiaging dietary interventions (90, 91), indicating that LP intake may induce heterogeneous biological effects. As discussed earlier, the evaluation of the ideal protein level for optimal effect on the health maintenance is complex and needs thus the evaluation of many health outcomes (80).

### LP and HP Diet during Gestation, Lactation, and Perinatal Periods

The early-life period, starting even before birth, is a key determinant of adult health. Environmental exposure, particularly nutrition, has a programming effect on later metabolic health.

Low protein and HP intakes during gestation and lactation are commonly viewed as stressors that can lead to changes in the body composition of the offspring.

Thus, it has been shown that feeding LP maternal diet during both gestation and lactation, or only during lactation, decreases the body weight and adiposity in both males and females (92, 93). By contrast, protein restriction only during gestation has no effect on males but leads to a lower LBM and higher body fat mass in females (93).

Interestingly, feeding pregnant rats with a LP diet results in a preference for high-fat foods in the offspring at the age of 12 weeks (94). In the same study, the authors reported that females, but not males, failed to adjust their energy intake and exhibited a higher adiposity. In line with these results, it was shown that a maternal LP diet results in low birth weight and subsequent adipose tissue catch-up growth when the offspring is fed a high-fat diet in male rats (95). Taken together, these results suggest that the exposition to a LP diet result in low birth weight and predispose to obesity when exposed to a high fat diet during the postnatal period.

Infant formulas have a higher protein content than breast milk, and the subsequent increase in protein intake of infants consuming formulas has been associated with increased risk of obesity (96). Thus, the impact of HP intake during the perinatal period needs further studies.

The effects of HP diet during gestation and lactation in animal models are controversial, leading to either no change or birth weight decrease (97–99). Likewise, HP diets can induce increase or decrease of body weight and adiposity (97, 100), depending on the experimental design.

High protein diets during gestation have been associated with higher adiposity and decreased energy expenditure in young male rats (101). Sex-specific effects of HP diets during both gestation and lactation predispose females, but not males, to higher body weight and adiposity (99, 102).

The protein source ingested by the mother during gestation and lactation can also influence body composition. Thus, it has been reported that when the maternal diet include soy protein, the offspring exhibit a higher body weight and adiposity compared to the offspring of dams fed with a casein-based diet. This is probably due to an alteration of food intake regulation in the offspring of dams fed a soy protein-based diet (103). Further experiments, including epigenetic modification measurement, are needed in order to decipher the underlying mechanisms explaining these latter results.

## Impact of Estrogen Deficiency on Body Weight and Physiological/Metabolic Parameters: Impact of Dietary Protein

**Figure 2:**
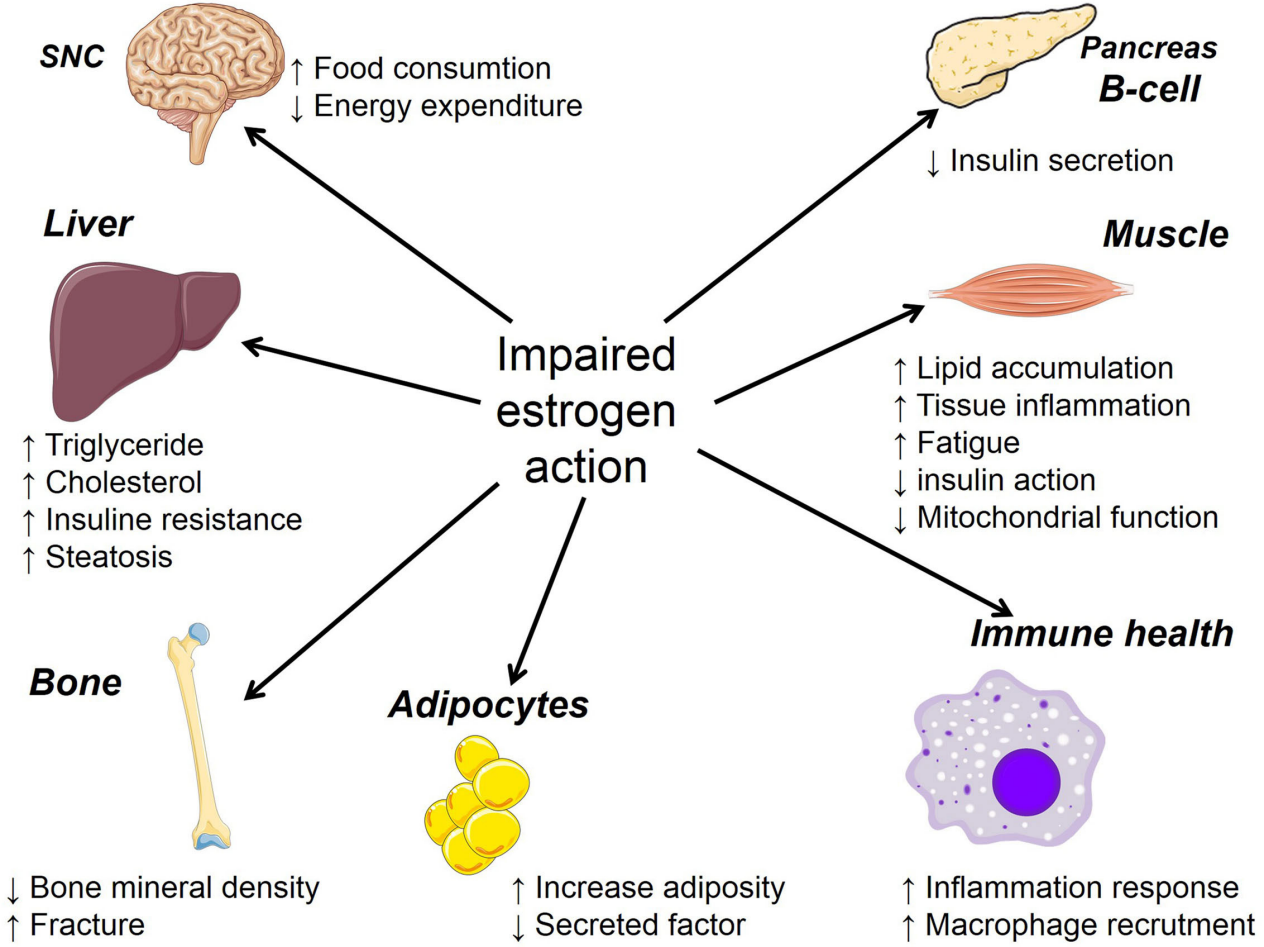
**Summary of the consequence of impaired estrogen action on the physiology of many target organs**.

The prevalence of metabolic syndrome, a constellation of abnormalities that includes obesity, hypertension, glucose intolerance, and dyslipidemia is higher in men than in women, but according to some epidemiological studies, this gender difference disappears after menopause (104). Animal studies have also reported protection of female mice from development of diet-induced obesity compared to age-matched males (105). However, as mentioned earlier, this advantage is lost in women at menopause, and the estrogen level decline is associated with central adiposity, insulin resistance, decreased energy expenditure, and greater risk of cardiovascular diseases (106, 107). Estrogen withdrawal during menopause is also associated with increased production of pro-inflammatory cytokines that are involved in many different diseases including osteoporosis, rheumatoid arthritis, and multiple myeloma (108). Figure 2 summarizes estrogen actions in the brain, adipose tissue, pancreatic islets, skeletal muscles, bone, liver, and macrophages, indicating the impact of estrogen on many tissues. These latter are known to act in synergy to promote glucose and lipid homeostasis.

As it is difficult, for obvious ethical reasons, to study in depth physiological and metabolic consequences of menopause in women, notably in a mechanistic perspective, surgical ovariectomy in animal models has been used to mimic estrogen deficiency. Ovariectomized (OVX) animal models have been widely used to study metabolic disorders associated with decrease of estradiol secretion in order to evaluate pharmacological and nutritional treatments that can safely reduce the consequences of menopause. Indeed, the use of OVX rats or mice to simulate the postmenopausal conditions is well established and represents a reproducible model. Notably, OVX animal models mimic metabolic modifications related to estrogen deficiency over a relatively short period of time. Witte et al. (109) showed that female rats and female mice do not have similar metabolic and behavioral responses after ovariectomy, demonstrating species differences in this experimental model. The OVX-induced weight gain in rats is mediated both by hyperphagia and reduced locomotor activity, while in mice the OVX procedure reduced locomotor activity and metabolic rate. Such species differences in response to OVX need to be taken into account when results are tentatively extrapolated to humans.

The most commonly used mice strain used to mimic consequences of estrogen withdrawal are C57BL/6J mice and C3H/Hen mice. One of the main consequences of the OVX procedure is an increase in the body weight. Incidentally, those models have been setup to study not only consequences of estrogen deficiency on obesity but also on various diseases including cancer, osteoporosis, cardiovascular diseases, and inflammation. Using the OVX C3H/Hen mice model to induce bone loss, studies (88, 89) have shown that even when the surgery was performed at 3 or 6 months, the procedure induced an increase in body weight related to an increase in the visceral and subcutaneous fat mass. However, when the OVX procedure was performed at 6 months, reduction of uterus weight was not observed compared to Sham mice. The spontaneous uterine atrophy observed in older Sham mice explains the absence of measurable difference. In the same study, the effect of raloxifene, a drug commonly indicated for osteoporosis which activates estrogen receptor (ER), prevented bone loss and the increase in body weight and fat mass observed following the OVX procedure (Table 2). However, raloxifene supplementation was not able to inhibit uterus weight loss. The preventive effect of raloxifene on weight gain in OVX C3H/Hen mice is in agreement with a recent study showing, in another mouse model, that selective activation of ER positively regulates mice metabolism (110).

**Table 2:** **Effect of ovariectomy, hormone replacement, or lactoferrin supplementation in OXV mice on body composition and bone mineral density after 12 weeks**.

Groups				
	Sham	OVX	OVX + raloxifene	OVX + LF
Initial body weight (g)	25.14 ± 1.18	24.20 ± 1.43	24.32 ± 1.06	24.70 ± 2.06
Final body weight (g)	35.50 ± 2.34^a^	41.74 ± 2.49^b^	34.61 ± 2.86^a^	42.68 ± 3.39^b^
Weight gain (g)	10.36 ± 1.84^a^	17.54 ± 2.21^b^	10.29 ± 1.43^a^	17.98 ± 2.48^b^
Uterus (mg)	253 ± 41^a^	131 ± 29^b^	127 ± 28^b^	160 ± 28^b^
Fat mass (g)	7.23 ± 1.63^a^	12.32 ± 2.03^b^	8.25 ± 1.35^a^	12.54 ± 2.15^b^
Carcass (g)	11.37 ± 0.59	12.87 ± 1.17	11.84 ± 0.72	12.54 ± 1.57
BMD gain (mg/cm^2^)	13 ± 2	8 ± 3^a^	13 ± 2	13 ± 2

*The surgery was performed on 12-week-old C3H mice. Data are means ± SEM (*n* = 10). A one-way ANOVA followed by Bonferroni’s *post hoc* was performed. Means with different superscript letters (a, b) are significantly different (*p* < 0.05) according to a Bonferroni multiple comparison test [adapted from Ref. (88, 89, 111)]*.

Mutations of ER are correlated to different aspects of the metabolic syndrome. Reduced ERα levels in the adipose tissue of obese individuals compared to the non-obese counterparts support a role of estrogen signaling in the control of body weight (112). The impact of ER activation on metabolic dysfunction related to menopause has also been studied using mice strain with a specific deletion of the ERα. Those mice become obese, glucose intolerant, hyperinsulinemeic and have decreased energy expenditure, decreased locomotion, and increased secretion of pro-inflammatory factors. The fat mass increase is associated not only with a decrease of the energy expenditure and of fat oxidation but also with an elevation of the circulating inflammatory markers (113, 114). Consequences of ERα deletion or ovariectomy are similar, supporting that ERα regulates mice energy metabolism (113). Moreover, loss of ERα in the central nervous system has been shown to induce hyperphagia and to decrease energy expenditure (115). Estradiol (E2), the major biologically active form of estrogen, is also known to positively influence insulin action in mice (113). Moreover, estrogen protection of female mice from development of diet-induced obesity and insulin resistance compared to age-matched males has also been demonstrated (105). A recent study using the ovariectomized mice model has shown that stimulation of estradiol receptor prevents weight gain, insulin resistance, and improved systemic metabolism.

**Figure 3:**
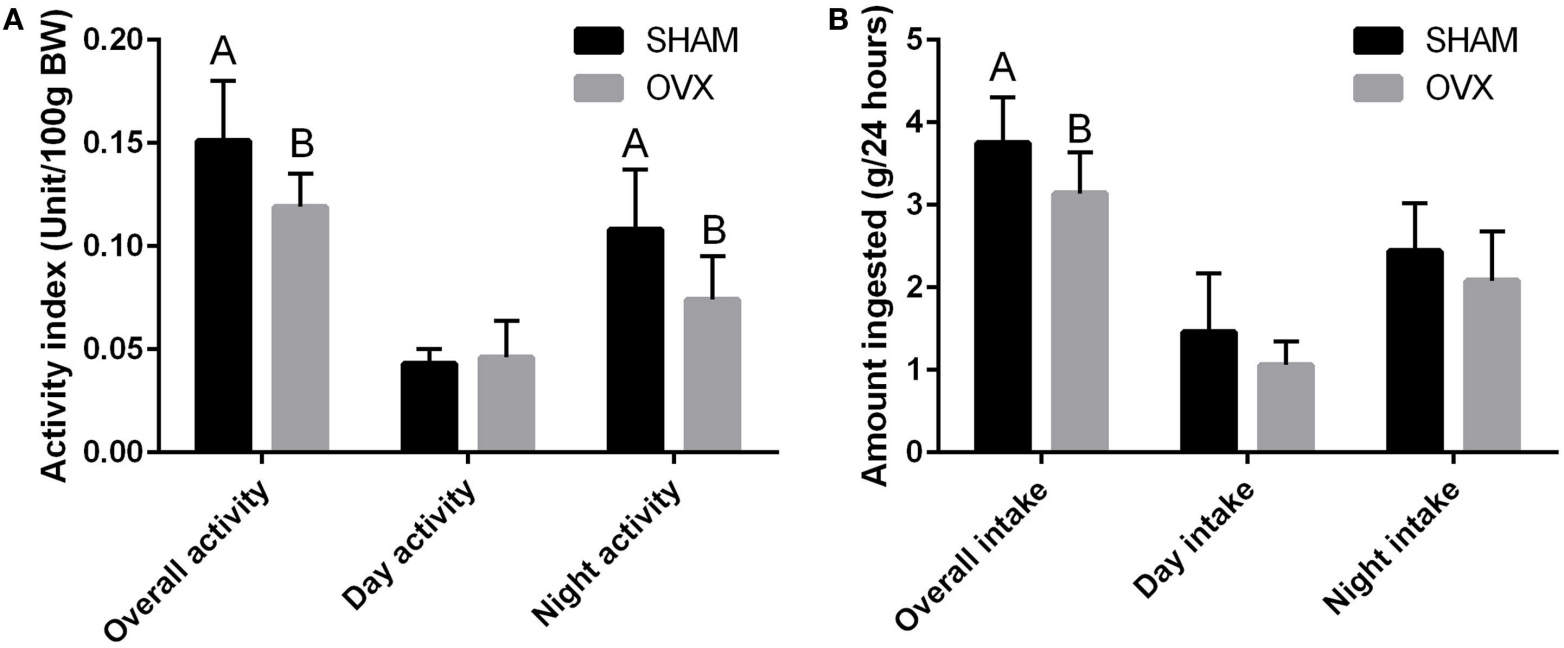
**Daily food intake analysis (A) and spontaneous physical activity (B) of Sham and OVX performed 10 weeks after the OVX procedure**. Data are means ± SEM (*n* = 8). Groups with different letters are significantly different (*p* < 0.05).

Food intake and spontaneous physical activity have been measured in OVX C3H/Hen mice, and both parameters were reduced compared to Sham mice (Figure 3). As the spontaneous physical activity reduction of the OVX mice is able to explain only a small part of the reduced ingestion, it is likely that the increased body weight and adiposity is related to a 15% decrease of the resting metabolism in the OVX mice. The absence of increased food intake and the reduced metabolic rate have also been reported in C57BL/6J mice (109). However, in mice lacking ERα in the central nervous system, a hyperphagia and decreased energy expenditure have been reported (115).

Moreover, using OVX C3H/Hen mice, it has been also possible to demonstrate that the OVX procedure is associated with immunological dysregulation. Indeed, Malet et al. (111) shown that estrogen deficiency induces heightened immune response sensitivity and an inflammatory status that were correlated to bone loss. Estrogen withdrawal is associated with T cell activation that produces essential osteoclastic factor such as RANKL and TNFα (108, 116). Lactoferrin (LF) ingestion has been shown to reduce T cell activation, pro-inflammatory cytokines, and consequently bone loss (111). Interestingly, LF is as efficient as raloxifene for the maintenance of bone mineral density in OVX mice, but did not reduce weight gain. However, neither compounds were able to preserve uterus weight (Table 2).

The menopause transition is associated not only with an increase in total body fat mass, visceral fat mass, and decreased energy expenditure but also with the increase of many inflammatory markers. Such an increase has indeed consequences on the incidence of many other pathologies including osteoporosis. However, obesity has been considered to have some beneficial effects for bone health in humans by some authors (117). The increase body weight and the ability of adipose tissue to synthesize estrogen support this proposition (118, 119). Since it has been proposed that estrogen synthesized by adipose tissue may have some antiresorptive effect on bone, Cao and Gregoire (120) studied the effect of high-fat diet on bone quality in OVX mice. This study shows that OVX mice fed with a high-fat diet, gain more weight, and had a higher estradiol level than mice feed with a standard diet, raising the question of the origin of estradiol production. However, the high-fat diet was not able to mitigate the OVX-induced bone loss in mice. Then, authors proposed that estrogen, likely synthesized by adipose tissue, does not have the same antiresorptive effect on bone as estrogen secreted by ovaries.

Those studies indicate that many non-elucidated mechanisms are involved in energy homeostasis in OVX mice. However, as OVX mice have a reduced energy expenditure similar to the one observed in estrogen-deficient women, it appears that mice is a useful model in that topic. Regarding the effect of dietary protein on dysfunctions related to ovariectomy, there is a relative paucity of available data. It has been shown that supplementation with water-insoluble fish protein has a cholesterol lowering effect in ovariectomized rats (121). Another dietary protein source, that is soybean extract, has been found to modulate the level of serum TGs in ovariectomized rats fed a cholesterolemic diet (122).

## The Pig Model for Research on Obesity

Regarding extrapolation to human situations, it is worth noting that the pig model is often considered as a model closer to humans than rodents for several aspects of physiological and metabolic studies. Indeed, the pig model has emerged as a relevant non-primate experimental animal for extrapolation to humans because of numerous similarities regarding anatomy, development, nutrition, and physiology (123–126). Pigs are also an animal model that is truly omnivorous, which make spontaneously individual meals, and which display striking similarities with humans in terms of nutritional requirements (127). It is also worth noting that the gut in the newborn pigs, although more mature that in newborn rodents, is, however, less mature than in infants (128). Another advantage of the pig model is that it is possible to recover a large number of cells (for instance, absorptive intestinal cells from both the small and large intestine after dietary intervention), even in young animals, in order to measure the impact of such intervention on cell metabolism and physiology (129, 130). In the pig model, preterm delivery at 90% gestation is comparable to preterm infant born at approximately 75% gestation (30 weeks) (131), making the pig neonate an interesting model for pediatric studies. In contrast to the rodent models, the size of newborn pig easily allows for tissue sampling and experimental manipulations of nutritional, physiological, and metabolic conditions. Finally, the pig model allows multi-catheterization and blood sampling without anemia thus allowing kinetics experiments (132).

However, even if the mini pig models, although relatively expensive, are increasingly used for research projects notably due to their limited size compared to regular farm pigs, the pig model present some drawbacks since it requires extensive areas for breeding, and is a source of abundant polluting compounds in biological fluids (fecal matter and urine), a situation incompatible with the use of the pig model in urban areas. As a matter of fact, in PubMed, the number of articles related to pig and obesity (798 articles) represents only about 2% of the number of articles related to rats/mice and obesity (35,318 articles). The readers are invited to refer to recent reviews regarding the pig model used for studies regarding the genetics of adiposity (133), the obese type 2 diabetes (134), the dietary modulation of gut microbiota and possible impact on obesity (135), the gastrointestinal hormones for eating control (136), and finally the establishment of food preferences and aversions (123) since these aspects will not be developed in this review. Regarding the specific aspect of the impact of the quantity and quality of dietary proteins on the gastrointestinal health in pigs, it has been shown that intestinal fermentation of the proteins results in the production of various potentially deleterious luminal products, which is often associated with growth of potential pathogens. In fact, excessive dietary protein intake (that mimic HP slimming diet) has been shown to stimulate in the pig model the growth of *Clostridium perfringens* and to reduce fecal counts of beneficial *Bifidobacteria* (137).

An increasing number of studies in pigs indicates that the gastrointestinal health is influenced by both the composition of the intestinal microbiota and its metabolic activity (138), this latter being impacted by the dietary composition, notably in terms of quantity and quality of dietary proteins (139). Indeed, the protein digestibility and protein AA composition are parameters that impact the profile of AA-derived bacterial metabolites in the large intestine. The use of fermentable carbohydrates to reduce deleterious protein-derived bacterial metabolites in pigs is well established (140), and for instance, soybean oligosaccharides have been shown to increase the presumably beneficial short-chain fatty acids while decreasing the protein-derived catabolites in the intestinal luminal content in weaned piglets (141). Last, interesting data have been recently obtained regarding the impact of the amount of dietary protein consumed by pigs on parameters like expression of AA and peptide transporters (125), or signaling pathways related to protein synthesis in muscles (142). Then, from these examples, it appears that using pig models for confirmation of data obtained in rodents represents a useful experimental strategy before further development of clinical studies implying dietary intervention with human volunteers, notably in overweight and obese individuals.

## Conclusion and Perspectives

Animal models are necessary in order to understand the mechanisms underlying the various biological parameters involved in the risk of obesity. Even if it is recognized that obesity results primarily from higher long-term energy consumption than energy expenditure, notably in case of low level of physical activity, we present here two situations in which animal models have been useful to understand how dietary (quantity and quality of dietary proteins) and physiological (menopause) modifications can impact parameters closely related to the development of obesity including body composition, food intake, energy expenditure, and tissue metabolism and physiology. Future research, notably in terms of mechanisms of action, using relevant animal models on the impact of dietary modifications at the different periods of age (notably during gestation, lactation, and perinatal periods of life) should allow to better enlighten on the best strategy for limiting the risk of obesity in young and aging adults.

## Author Contributions

C-DT and BA wrote the manuscript that was improved by BF and TD.

## Conflict of Interest Statement

The authors declare that the research was conducted in the absence of any commercial or financial relationships that could be construed as a potential conflict of interest.

## References

[1] Seidell JC Obesity in Europe. Obes Res (1995) 3:89s–93s. 10.1002/j.1550-8528.1995.tb00451.x 8581793

[2] Bahreini N Noor MI Koon PB Talib RA Lubis SH Ganjali M Weight status among Iranian adolescents: comparison of four different criteria. J Res Med Sci (2013) 18:641–6. 24379838 PMC3872601

[3] Rubio-Aliaga I. Model organisms in molecular nutrition research. Mol Nutr Food Res (2012) 56:844–53. 10.1002/mnfr.201100784 22707260

[4] Pesta DH Samuel VT. A high-protein diet for reducing body fat: mechanisms and possible caveats. Nutr Metab (2014) 11:1–8. 10.1186/1743-7075-11-53 25489333 PMC4258944

[5] Arentson-Lantz E Clairmont S Paddon-Jones D Tremblay A Elango R. Protein: a nutrient in focus. Appl Physiol Nutr Metab (2015) 40:755–61. 10.1139/apnm-2014-0530 26197807

[6] Houston DK Nicklas BJ Ding J Harris TB Tylavsky FA Newman AB Dietary protein intake is associated with lean mass change in older, community-dwelling adults: the Health, Aging, and Body Composition (Health ABC) Study. Am J Clin Nutr (2008) 87:150–5. 18175749 10.1093/ajcn/87.1.150

[7] Wilson J Wilson GJ. Contemporary issues in protein requirements and consumption for resistance trained athletes. J Int Soc Sports Nutr (2006) 3:1. 10.1186/1550-2783-3-1-7 18500966 PMC2129150

[8] Menaker L Navia JM Appetite regulation in the rat under various physiological conditions: the role of dietary protein and calories. J Nutr (1973) 103:347–52. 4688147 10.1093/jn/103.3.347

[9] Sørensen A Mayntz D Raubenheimer D Simpson SJ. Protein-leverage in mice: the geometry of macronutrient balancing and consequences for fat deposition. Obesity (2008) 16:566–71. 10.1038/oby.2007.58 18239565

[10] Simpson SJ Raubenheimer D. Obesity: the protein leverage hypothesis. Obes Rev (2005) 6:133–42. 10.1111/j.1467-789X.2005.00178.x 15836464

[11] Blouet C Mariotti F Azzout-Marniche D Bos C Mathé V Tomé D The reduced energy intake of rats fed a high-protein low-carbohydrate diet explains the lower fat deposition, but macronutrient substitution accounts for the improved glycemic control. J Nutr (2006) 136:1849–54. 16772448 10.1093/jn/136.7.1849

[12] Jean C Rome S Mathé V Huneau J-F Aattouri N Fromentin G Metabolic evidence for adaptation to a high protein diet in rats. J Nutr (2001) 131:91–8. 11208943 10.1093/jn/131.1.91

[13] Aparecida de França S dos Santos MP Garófalo MAR Navegantes LC Kettelhut I do C Lopes CF Low protein diet changes the energetic balance and sympathetic activity in brown adipose tissue of growing rats. Nutrition (2009) 25:1186–92. 10.1016/j.nut.2009.03.011 19535223

[14] Huang X Hancock DP Gosby AK McMahon AC Solon SMC Le Couteur DG Effects of dietary protein to carbohydrate balance on energy intake, fat storage, and heat production in mice. Obesity (2013) 21:85–92. 10.1002/oby.20007 23404943

[15] Solon-Biet SM McMahon AC Ballard JWO Ruohonen K Wu LE Cogger VC The ratio of macronutrients, not caloric intake, dictates cardiometabolic health, aging, and longevity in ad libitum-fed mice. Cell Metab (2014) 19:418–30. 10.1016/j.cmet.2014.02.009 24606899 PMC5087279

[16] Dubuisson C Lioret S Touvier M Dufour A Calamassi-Tran G Volatier J-L Trends in food and nutritional intakes of French adults from 1999 to 2007: results from the INCA surveys. Br J Nutr (2010) 103:1035. 10.1017/S0007114509992625 20028601

[17] Eisenstein J Roberts SB Dallal G Saltzman E. High-protein weight-loss diets: are they safe and do they work? A review of the experimental and epidemiologic data. Nutr Rev (2002) 60:189–200. 10.1301/00296640260184264 12144197

[18] Blachier F Mariotti F Huneau JF Tomé D. Effects of amino acid-derived luminal metabolites on the colonic epithelium and physiopathological consequences. Amino Acids (2007) 33:547–62. 10.1007/s00726-006-0477-9 17146590

[19] Shoda R Matsueda K Yamato S Umeda N. Epidemiologic analysis of Crohn disease in Japan: increased dietary intake of n-6 polyunsaturated fatty acids and animal protein relates to the increased incidence of Crohn disease in Japan. Am J Clin Nutr (1996) 63:741–5. 8615358 10.1093/ajcn/63.5.741

[20] Jantchou P Morois S Clavel-Chapelon F Boutron-Ruault M-C Carbonnel F. Animal protein intake and risk of inflammatory bowel disease: the E3N prospective study. Am J Gastroenterol (2010) 105:2195–201. 10.1038/ajg.2010.192 20461067

[21] Spooren CEGM Pierik MJ Zeegers MP Feskens EJM Masclee AAM Jonkers DMAE. Review article: the association of diet with onset and relapse in patients with inflammatory bowel disease. Aliment Pharmacol Ther (2013) 38:1172–87. 10.1111/apt.12501 24118051

[22] Portune KJ Beaumont M Davila A-M Tomé D Blachier F Sanz Y Gut microbiota role in dietary protein metabolism and health-related outcomes: the two sides of the coin. Trends Food Sci Technol (2016) 57:213–32. 10.1016/j.tifs.2016.08.011

[23] Lagiou P Sandin S Weiderpass E Lagiou A Mucci L Trichopoulos D Low carbohydrate/high protein diet and mortality in a cohort of Swedish women. J Intern Med (2007) 261:366–74. 10.1111/j.1365-2796.2007.01774.x 17391111

[24] Lagiou P Sandin S Lof M Trichopoulos D Adami H-O Weiderpass E. Low carbohydrate-high protein diet and incidence of cardiovascular diseases in Swedish women: prospective cohort study. BMJ (2012) 344:e4026. 10.1136/bmj.e4026 22735105 PMC3383863

[25] MacLeod EL Ney DM Prise en charge nutritionnelle de la phénylcétonurie. Ann Nestlé Ed Fr (2010) 68:60–71. 10.1159/000323156

[26] Mandayam S Mitch WE Dietary protein restriction benefits patients with chronic kidney disease (review article). Nephrology (2006) 11:53–7. 10.1111/j.1440-1797.2006.00528.x 16509933

[27] Patel MS Srinivasan M Metabolic programming in the immediate postnatal life. Ann Nutr Metab (2011) 58:18–28. 10.1159/000328040 21846978 PMC3190171

[28] Steyn NP Mchiza Z Hill J Davids YD Venter I Hinrichsen E Nutritional contribution of street foods to the diet of people in developing countries: a systematic review. Public Health Nutr (2014) 17:1363–74. 10.1017/S1368980013001158 23680029 PMC10282211

[29] Andriamihaja M Davila A-M Eklou-Lawson M Petit N Delpal S Allek F Colon luminal content and epithelial cell morphology are markedly modified in rats fed with a high-protein diet. Am J Physiol Gastrointest Liver Physiol (2010) 299:G1030–7. 10.1152/ajpgi.00149.2010 20689060

[30] Lacroix M Gaudichon C Martin A Morens C Mathé V Tomé D A long-term high-protein diet markedly reduces adipose tissue without major side effects in Wistar male rats. Am J Physiol Regul Integr Comp Physiol (2004) 287:R934–42. 10.1152/ajpregu.00100.2004 15155276

[31] Blouet C Ono H Schwartz GJ. Mediobasal hypothalamic p70 S6 kinase 1 modulates the control of energy homeostasis. Cell Metab (2008) 8:459–67. 10.1016/j.cmet.2008.10.004 19041762 PMC2637401

[32] Pichon L Huneau J-F Fromentin G Tomé D. A high-protein, high-fat, carbohydrate-free diet reduces energy intake, hepatic lipogenesis, and adiposity in rats. J Nutr (2006) 136:1256–60. 16614413 10.1093/jn/136.5.1256

[33] Mellinkoff S Frankland M Relationship between serum amino acid concentration and fluctuations in appetite. J Appl Physiol (1956) 8:535–8. 13295170 10.1152/jappl.1956.8.5.535

[34] Arrieta-Cruz I Gutierrez-Juarez R. The role of circulating amino acids in the hypothalamic regulation of liver glucose metabolism. Adv Nutr (2016) 7:790S–7S. 10.3945/an.115.011171 27422516 PMC4942863

[35] Lam DD Garfield AS Marston OJ Shaw J Heisler LK. Brain serotonin system in the coordination of food intake and body weight. Pharmacol Biochem Behav (2010) 97:84–91. 10.1016/j.pbb.2010.09.003 20837046

[36] Nakajima S Tanaka N Hamada M Tsuchiya T Okuda H Correlation between energy and histidine intake in female living in Setouchi area. J Jpn Soc Stud Obes (2001) 7:276–82.

[37] Bensaıd A Tomé D L’Heureux-Bourdon D Even P Gietzen D Morens C A high-protein diet enhances satiety without conditioned taste aversion in the rat. Physiol Behav (2003) 78:311–20. 10.1016/S0031-9384(02)00977-0 12576130

[38] Bensaïd A Tomé D Gietzen D Even P Morens C Gausseres N Protein is more potent than carbohydrate for reducing appetite in rats. Physiol Behav (2002) 75:577–82. 10.1016/S0031-9384(02)00646-7 12062322

[39] Du F Higginbotham DA White BD. Food intake, energy balance and serum leptin concentrations in rats fed low-protein diets. J Nutr (2000) 130:514–21. 10702578 10.1093/jn/130.3.514

[40] Morrison CD Reed SD Henagan TM. Homeostatic regulation of protein intake: in search of a mechanism. Am J Physiol Regul Integr Comp Physiol (2012) 302:R917–28. 10.1152/ajpregu.00609.2011 22319049 PMC3330767

[41] Batterham RL Heffron H Kapoor S Chivers JE Chandarana K Herzog H Critical role for peptide YY in protein-mediated satiation and body-weight regulation. Cell Metab (2006) 4:223–33. 10.1016/j.cmet.2006.08.001 16950139

[42] Morrison C Laeger T. Protein-dependent regulation of feeding and metabolism. Trends Endocrinol Metab (2015) 26:256–62. 10.1016/j.tem.2015.02.008 25771038 PMC4416985

[43] Laeger T Henagan TM Albarado DC Redman LM Bray GA Noland RC FGF21 is an endocrine signal of protein restriction. J Clin Invest (2014) 124:3913–22. 10.1172/JCI74915 25133427 PMC4153701

[44] Kasaoka S Tsuboyama-Kasaoka N Kawahara Y Inoue S Tsuji M Ezaki O Histidine supplementation suppresses food intake and fat accumulation in rats. Nutrition (2004) 20:991–6. 10.1016/j.nut.2004.08.006 15561489

[45] Endo M Kasaoka S Takizawa M Goto K Nakajima S Moon S-K Suppressed fat accumulation in rats fed a histidine-enriched diet. J Food Sci Nutr (2010) 15:1–6. 10.3746/jfn.2010.15.1.001

[46] Freudenberg A Petzke KJ Klaus S Dietary l-leucine and l-alanine supplementation have similar acute effects in the prevention of high-fat diet-induced obesity. Amino Acids (2012) 44:519–28. 10.1007/s00726-012-1363-2 22847780

[47] Ruan Z Yang Y Wen Y Zhou Y Fu X Ding S Metabolomic analysis of amino acid and fat metabolism in rats with l-tryptophan supplementation. Amino Acids (2014) 46(12):2681–91. 10.1007/s00726-014-1823-y 25139634

[48] Jobgen W Meininger CJ Jobgen SC Li P Lee M-J Smith SB Dietary l-arginine supplementation reduces white fat gain and enhances skeletal muscle and brown fat masses in diet-induced obese rats. J Nutr (2008) 139:230–7. 10.3945/jn.108.096362 19106310 PMC3151442

[49] Block K Harper A High levels of dietary amino and branched-chain α-keto acids alter plasma and brain amino acid concentrations in rats. J Nutr (1991) 121:663–71. 2019876 10.1093/jn/121.5.663

[50] Badole SL Bagul PP Mahamuni SP Khose RD Joshi AC Jangam GB Oral l-glutamine increases active GLP-1 (7-36) amide secretion and improves glycemic control in stretpozotocin-nicotinamide induced diabetic rats. Chem Biol Interact (2013) 203:530–41. 10.1016/j.cbi.2013.02.006 23466488

[51] Clemmensen C Smajilovic S Smith EP Woods SC Bräuner-Osborne H Seeley RJ Oral l-arginine stimulates GLP-1 secretion to improve glucose tolerance in male mice. Endocrinology (2013) 154:3978–83. 10.1210/en.2013-1529 23959939 PMC3800753

[52] Zhang Y Guo K LeBlanc RE Loh D Schwartz GJ Yu Y-H. Increasing dietary leucine intake reduces diet-induced obesity and improves glucose and cholesterol metabolism in mice via multimechanisms. Diabetes (2007) 56:1647–54. 10.2337/db07-0123 17360978

[53] Cota D Proulx K Smith KA Kozma SC Thomas G Woods SC Hypothalamic mTOR signaling regulates food intake. Science (2006) 312:927–30. 10.1126/science.1124147 16690869

[54] Morrison CD Xi X White CL Ye J Martin RJ. Amino acids inhibit Agrp gene expression via an mTOR-dependent mechanism. Am J Physiol Endocrinol Metab (2007) 293:E165–71. 10.1152/ajpendo.00675.2006 17374702 PMC2596875

[55] Ropelle ER Pauli JR Fernandes MFA Rocco SA Marin RM Morari J A central role for neuronal AMP-activated protein kinase (AMPK) and mammalian target of rapamycin (mTOR) in high-protein diet-induced weight loss. Diabetes (2008) 57:594–605. 10.2337/db07-0573 18057094

[56] Menezes AL Pereira MP Buzelle SL dos Santos MP de França SA Baviera AM A low-protein, high-carbohydrate diet increases de novo fatty acid synthesis from glycerol and glycerokinase content in the liver of growing rats. Nutr Res (2013) 33:494–502. 10.1016/j.nutres.2013.04.010 23746566

[57] Henagan TM Laeger T Navard AM Albarado D Noland RC Stadler K Hepatic autophagy contributes to the metabolic response to dietary protein restriction. Metabolism (2016) 65:805–15. 10.1016/j.metabol.2016.02.015 27173459 PMC4867053

[58] Buzelle SL Santos MP Baviera AM Lopes CF Garófalo MAR Navegantes LCC A low-protein, high-carbohydrate diet increases the adipose lipid content without increasing the glycerol-3-phosphate or fatty acid content in growing rats. Can J Physiol Pharmacol (2010) 88:1157–65. 10.1139/Y10-096 21164562

[59] Stepien M Gaudichon C Azzout-Marniche D Fromentin G Tome D Even P Postprandial nutrient partitioning but not energy expenditure is modified in growing rats during adaptation to a high-protein diet. J Nutr (2010) 140:939–45. 10.3945/jn.109.120139 20335631

[60] Stepien M Gaudichon C Fromentin G Even P Tomé D Azzout-Marniche D. Increasing protein at the expense of carbohydrate in the diet down-regulates glucose utilization as glucose sparing effect in rats. PLoS One (2011) 6:e14664. 10.1371/journal.pone.0014664 21326875 PMC3034717

[61] Fromentin C Azzout-Marniche D Tomé D Even P Luengo C Piedcoq J The postprandial use of dietary amino acids as an energy substrate is delayed after the deamination process in rats adapted for 2 weeks to a high protein diet. Amino Acids (2010) 40:1461–72. 10.1007/s00726-010-0756-3 20890620

[62] Blachier F Mourtada A Sener A Malaisse WJ Stimulus-secretion coupling of arginine-induced insulin release. Uptake of metabolized and nonmetabolized cationic amino acids by pancreatic islets*. Endocrinology (1989) 124:134–41. 10.1210/endo-124-1-134 2462484

[63] Sener A Blachier F Rasschaert J Mourtada A Malaisse-Lagae F Malaisse WJ Stimulus-secretion coupling of arginine-induced insulin release: comparison with lysine-induced insulin secretion*. Endocrinology (1989) 124:2558–67. 10.1210/endo-124-5-2558 2495931

[64] Veldhorst MAB Westerterp KR Westerterp-Plantenga MS. Gluconeogenesis and protein-induced satiety. Br J Nutr (2012) 107:595–600. 10.1017/S0007114511003254 21767449

[65] Azzout-Marniche D Gaudichon C Blouet C Bos C Mathe V Huneau J-F Liver glyconeogenesis: a pathway to cope with postprandial amino acid excess in high-protein fed rats? Am J Physiol Regul Integr Comp Physiol (2006) 292:R1400–7. 10.1152/ajpregu.00566.2006 17158265

[66] Baum JI Layman DK Freund GG Rahn KA Nakamura MT Yudell BE. A reduced carbohydrate, increased protein diet stabilizes glycemic control and minimizes adipose tissue glucose disposal in rats. J Nutr (2006) 136:1855–61. 16772449 10.1093/jn/136.7.1855

[67] Obeid OA Boukarim LK Al Awar RM Hwalla N. Postprandial glycogen and lipid synthesis in prednisolone-treated rats maintained on high-protein diets with varied carbohydrate levels. Nutrition (2006) 22:288–94. 10.1016/j.nut.2005.07.014 16412611

[68] Acheson K Influence of auonomic nervous system on nutrient-induced thermogenesis in humans. Nutrition (1993) 9:373–80. 8400596

[69] Westerterp KR Diet induced thermogenesis. Nutr Metab (2004) 1:1 10.1186/1743-7075-1-1 PMC524030 15507147

[70] Mikkelsen PB Toubro S Astrup A. Effect of fat-reduced diets on 24-h energy expenditure: comparisons between animal protein, vegetable protein, and carbohydrate. Am J Clin Nutr (2000) 72:1135–41. 11063440 10.1093/ajcn/72.5.1135

[71] Even P Nicolaidis S. Spontaneous and 2DG induced metabolic changes and feeding: the ischymetric hypothesis. Brain Res Bull (1985) 15:429–35. 10.1016/0361-9230(85)90012-7 4063836

[72] Friedman M Control of energy intake by energy metabolism. Am J Clin Nutr (1995) 62:1096–100. 10.1093/ajcn/62.5.1096S 7484927

[73] Chalvon-Demersay T Even PC Tomé D Chaumontet C Piedcoq J Gaudichon C Low-protein diet induces, whereas high-protein diet reduces hepatic FGF21 production in mice, but glucose and not amino acids up-regulate FGF21 in cultured hepatocytes. J Nutr Biochem (2016) 36:60–7. 10.1016/j.jnutbio.2016.07.002 27574977

[74] Laeger T Albarado DC Burke SJ Trosclair L Hedgepeth JW Berthoud H-R Metabolic responses to dietary protein restriction require an increase in FGF21 that is delayed by the absence of GCN2. Cell Rep (2016) 16:707–16. 10.1016/j.celrep.2016.06.044 27396336 PMC4956501

[75] Petzke KJ Riese C Klaus S. Short-term, increasing dietary protein and fat moderately affect energy expenditure, substrate oxidation and uncoupling protein gene expression in rats. J Nutr Biochem (2007) 18:400–7. 10.1016/j.jnutbio.2006.07.005 16979329

[76] de França SA dos Santos MP Przygodda F Garófalo MAR Kettelhut IC Magalhães DA A low-protein, high-carbohydrate diet stimulates thermogenesis in the brown adipose tissue of rats via ATF-2. Lipids (2016) 51:303–10. 10.1007/s11745-016-4119-z 26781764

[77] Malloy VL Krajcik RA Bailey SJ Hristopoulos G Plummer JD Orentreich N. Methionine restriction decreases visceral fat mass and preserves insulin action in aging male Fischer 344 rats independent of energy restriction. Aging Cell (2006) 5:305–14. 10.1111/j.1474-9726.2006.00220.x 16800846

[78] Hasek BE Stewart LK Henagan TM Boudreau A Lenard NR Black C Dietary methionine restriction enhances metabolic flexibility and increases uncoupled respiration in both fed and fasted states. Am J Physiol Regul Integr Comp Physiol (2010) 299:R728–39. 10.1152/ajpregu.00837.2009 20538896 PMC2944433

[79] Hasek BE Boudreau A Shin J Feng D Hulver M Van NT Remodeling the integration of lipid metabolism between liver and adipose tissue by dietary methionine restriction in rats. Diabetes (2013) 62:3362–72. 10.2337/db13-0501 23801581 PMC3781441

[80] Wolfe RR. Update on protein intake: importance of milk proteins for health status of the elderly. Nutr Rev (2015) 73:41–7. 10.1093/nutrit/nuv021 26175489 PMC4597363

[81] Norton LE Wilson GJ Layman DK Moulton CJ Garlick PJ Protein distribution affects muscle mass based on differences in postprandial muscle protein synthesis and plasma leucine in rats. J Int Soc Sports Nutr (2012) 9:23 10.1186/1550-2783-9-S1-P2322607394

[82] Le Couteur DG Solon-Biet S Cogger VC Mitchell SJ Senior A de Cabo R The impact of low-protein high-carbohydrate diets on aging and lifespan. Cell Mol Life Sci (2016) 73:1237–52. 10.1007/s00018-015-2120-y 26718486 PMC11108352

[83] Sun L Sadighi Akha AA Miller RA Harper JM. Life-span extension in mice by preweaning food restriction and by methionine restriction in middle age. J Gerontol A Biol Sci Med Sci (2009) 64A:711–22. 10.1093/gerona/glp051 19414512 PMC2691799

[84] Orentreich N Matias JR DeFelice A Zimmerman J. Low methionine ingestion by rats extends life span. Age Days (1993) 1050:1300. 8429371 10.1093/jn/123.2.269

[85] Miller RA Buehner G Chang Y Harper JM Sigler R Smith-Wheelock M Methionine-deficient diet extends mouse lifespan, slows immune and lens aging, alters glucose, T4, IGF-I and insulin levels, and increases hepatocyte MIF levels and stress resistance: methionine restriction slows mouse aging. Aging Cell (2005) 4:119–25. 10.1111/j.1474-9726.2005.00152.x 15924568 PMC7159399

[86] Rouy E Vico L Laroche N Benoit V Rousseau B Blachier F Protein quality affects bone status during moderate protein restriction in growing mice. Bone (2014) 59:7–13. 10.1016/j.bone.2013.10.013 24495359

[87] Ables GP Perrone CE Orentreich D Orentreich N. Methionine-restricted C57BL/6J mice are resistant to diet-induced obesity and insulin resistance but have low bone density. PLoS One (2012) 7:e51357. 10.1371/journal.pone.0051357 23236485 PMC3518083

[88] Guillerminet F Beaupied H Fabien-Soulé V Tomé D Benhamou C-L Roux C Hydrolyzed collagen improves bone metabolism and biomechanical parameters in ovariectomized mice: an in vitro and in vivo study. Bone (2010) 46:827–34. 10.1016/j.bone.2009.10.035 19895915

[89] Guillerminet F Fabien-Soulé V Even PC Tomé D Benhamou C-L Roux C Hydrolyzed collagen improves bone status and prevents bone loss in ovariectomized C3H/HeN mice. Osteoporos Int (2012) 23:1909–19. 10.1007/s00198-011-1788-6 21927918

[90] Fontana L Weiss EP Villareal DT Klein S Holloszy JO. Long-term effects of calorie or protein restriction on serum IGF-1 and IGFBP-3 concentration in humans. Aging Cell (2008) 7:681–7. 10.1111/j.1474-9726.2008.00417.x 18843793 PMC2673798

[91] Levine ME Suarez JA Brandhorst S Balasubramanian P Cheng C-W Madia F Low protein intake is associated with a major reduction in IGF-1, cancer, and overall mortality in the 65 and younger but not older population. Cell Metab (2014) 19:407–17. 10.1016/j.cmet.2014.02.006 24606898 PMC3988204

[92] Fagundes ATS Moura EG Passos MCF Oliveira E Toste FP Bonomo IT Maternal low-protein diet during lactation programmes body composition and glucose homeostasis in the adult rat offspring. Br J Nutr (2007) 98:922–8. 10.1017/S0007114507750924 17524178

[93] Zambrano E Bautista CJ Deás M Martínez-Samayoa PM González-Zamorano M Ledesma H A low maternal protein diet during pregnancy and lactation has sex- and window of exposure-specific effects on offspring growth and food intake, glucose metabolism and serum leptin in the rat: maternal low protein alters offspring growth and metabolism. J Physiol (2006) 571:221–30. 10.1113/jphysiol.2005.100313 16339179 PMC1805642

[94] Bellinger L Lilley C Langley-Evans SC. Prenatal exposure to a maternal low-protein diet programmes a preference for high-fat foods in the young adult rat. Br J Nutr (2004) 92:513. 10.1079/BJN20041224 15469656

[95] Claycombe KJ Uthus EO Roemmich JN Johnson LK Johnson WT. Prenatal low-protein and postnatal high-fat diets induce rapid adipose tissue growth by inducing Igf2 expression in Sprague Dawley rat offspring. J Nutr (2013) 143:1533–9. 10.3945/jn.113.178038 23946348

[96] Weber M Grote V Closa-Monasterolo R Escribano J Langhendries J-P Dain E Lower protein content in infant formula reduces BMI and obesity risk at school age: follow-up of a randomized trial. Am J Clin Nutr (2014) 99:1041–51. 10.3945/ajcn.113.064071 24622805

[97] Desclee de Maredsous C Oozeer R Barbillon P Mary-Huard T Delteil C Blachier F High-protein exposure during gestation or lactation or after weaning has a period-specific signature on rat pup weight, adiposity, food intake, and glucose homeostasis up to 6 weeks of age. J Nutr (2016) 146:21–9. 10.3945/jn.115.216465 26674762

[98] Metges CC Gors S Lang IS Hammon HM Brussow K-P Weitzel JM Low and high dietary protein:carbohydrate ratios during pregnancy affect materno-fetal glucose metabolism in pigs. J Nutr (2014) 144:155–63. 10.3945/jn.113.182691 24353346

[99] Thone-Reineke C. High-protein nutrition during pregnancy and lactation programs blood pressure, food efficiency, and body weight of the offspring in a sex-dependent manner. Am J Physiol Regul Integr Comp Physiol (2006) 291:R1025–30. 10.1152/ajpregu.00898.2005 16675628

[100] Sarr O Gondret F Jamin A Le Huerou-Luron I Louveau I. A high-protein neonatal formula induces a temporary reduction of adiposity and changes later adipocyte physiology. Am J Physiol Regul Integr Comp Physiol (2011) 300:R387–97. 10.1152/ajpregu.00459.2010 21123765

[101] Daenzer M Ortmann S Klaus S Metges CC. Prenatal high protein exposure decreases energy expenditure and increases adiposity in young rats. J Nutr (2002) 132:142–4. 11823569 10.1093/jn/132.2.142

[102] Hallam MC Reimer RA. A maternal high-protein diet predisposes female offspring to increased fat mass in adulthood whereas a prebiotic fibre diet decreases fat mass in rats. Br J Nutr (2013) 110:1732–41. 10.1017/S0007114513000998 23561448

[103] Jahan-Mihan A Rodriguez J Christie C Sadeghi M Zerbe T. The role of maternal dietary proteins in development of metabolic syndrome in offspring. Nutrients (2015) 7:9185–217. 10.3390/nu7115460 26561832 PMC4663588

[104] Miranda PJ DeFronzo RA Califf RM Guyton JR Metabolic syndrome: definition, pathophysiology, and mechanisms. Am Heart J (2005) 149:33–45. 10.1016/j.ahj.2004.07.013 15660032

[105] Pettersson US Waldén TB Carlsson P-O Jansson L Phillipson M. Female mice are protected against high-fat diet induced metabolic syndrome and increase the regulatory T cell population in adipose tissue. PLoS One (2012) 7:e46057. 10.1371/journal.pone.0046057 23049932 PMC3458106

[106] Lovejoy JC Champagne CM de Jonge L Xie H Smith SR. Increased visceral fat and decreased energy expenditure during the menopausal transition. Int J Obes (2008) 32:949–58. 10.1038/ijo.2008.25 18332882 PMC2748330

[107] Wilson P Garrison R Castelli W. Postmenopausal estrogen use, cigarette smoking, and cardiovascular morbidity in women over 50. The Framingham Study. N Engl J Med (1985) 313:1038–43. 10.1056/NEJM198510243131702 2995808

[108] Mundy GR Osteoporosis and inflammation. Nutr Rev (2007) 65:147–51. 10.1301/nr.2007.dec.S147-S151 18240539

[109] Witte MM Resuehr D Chandler AR Mehle AK Overton JM. Female mice and rats exhibit species-specific metabolic and behavioral responses to ovariectomy. Gen Comp Endocrinol (2010) 166:520–8. 10.1016/j.ygcen.2010.01.006 20067798 PMC2856744

[110] Hamilton DJ Minze LJ Kumar T Cao TN Lyon CJ Geiger PC Estrogen receptor alpha activation enhances mitochondrial function and systemic metabolism in high-fat-fed ovariectomized mice. Physiol Rep (2016) 4:e12913. 10.14814/phy2.12913 27582063 PMC5027347

[111] Malet A Bournaud E Lan A Mikogami T Tomé D Blais A. Bovine lactoferrin improves bone status of ovariectomized mice via immune function modulation. Bone (2011) 48:1028–35. 10.1016/j.bone.2011.02.002 21303707

[112] Nilsson M Holst JJ Björck IM. Metabolic effects of amino acid mixtures and whey protein in healthy subjects: studies using glucose-equivalent drinks. Am J Clin Nutr (2007) 85:996–1004. 17413098 10.1093/ajcn/85.4.996

[113] Ribas V Nguyen MTA Henstridge DC Nguyen A-K Beaven SW Watt MJ Impaired oxidative metabolism and inflammation are associated with insulin resistance in ER-deficient mice. AJP Endocrinol Metab (2010) 298:E304–19. 10.1152/ajpendo.00504.2009 PMC2822483 19920214

[114] Ribas V Drew BG Le JA Soleymani T Daraei P Sitz D Myeloid-specific estrogen receptor deficiency impairs metabolic homeostasis and accelerates atherosclerotic lesion development. Proc Natl Acad Sci U S A (2011) 108:16457–62. 10.1073/pnas.1104533108 21900603 PMC3182726

[115] Xu Y Nedungadi TP Zhu L Sobhani N Irani BG Davis KE Distinct hypothalamic neurons mediate estrogenic effects on energy homeostasis and reproduction. Cell Metab (2011) 14:453–65. 10.1016/j.cmet.2011.08.009 21982706 PMC3235745

[116] Pacifici R Role of T cells in ovariectomy induced bone loss-revisited. J Bone Miner Res (2012) 27:231–9. 10.1002/jbmr.1500 22271394

[117] Villareal DT Apovian CM Kushner RF Klein S. Obesity in older adults: technical review and position statement of the American Society for Nutrition and NAASO, The Obesity Society. Obes Res (2005) 13:1849–63. 10.1038/oby.2005.228 16339115

[118] Reid CL Murgatroyd PR Wright A Menon DK. Quantification of lean and fat tissue repletion following critical illness: a case report. Crit Care (2008) 12:1. 10.1186/cc6929 18559097 PMC2481478

[119] Simpson E. Sources of estrogen and their importance. J Steroid Biochem Mol Biol (2003) 86:225–30. 10.1016/S0960-0760(03)00360-1 14623515

[120] Cao JJ Gregoire BR. A high-fat diet increases body weight and circulating estradiol concentrations but does not improve bone structural properties in ovariectomized mice. Nutr Res (2016) 36:320–7. 10.1016/j.nutres.2015.12.008 27001277

[121] Kato M Ogawa H Kishida T Ebihara K. The mechanism of the cholesterol-lowering effect of water-insoluble fish protein in ovariectomised rats. Br J Nutr (2009) 102:816. 10.1017/S0007114509316153 19335928

[122] Lee BH Lee HH Kim JH Cho BR Choi Y-S. Effects of a soluble fraction of soybean on lipid profiles in ovariectomized rats fed a cholesterolemic diet. J Med Food (2007) 10:521–5. 10.1089/jmf.2006.194 17887947

[123] Clouard C Meunier-Salaün M-C Val-Laillet D The effects of sensory functional ingredients on food preferences, intake and weight gain in juvenile pigs. Appl Anim Behav Sci (2012) 138:36–46. 10.1016/j.applanim.2012.01.016

[124] He Q Ren P Kong X Wu Y Wu G Li P Comparison of serum metabolite compositions between obese and lean growing pigs using an NMR-based metabonomic approach. J Nutr Biochem (2012) 23:133–9. 10.1016/j.jnutbio.2010.11.007 21429726

[125] Liu Y Kong X Li F Tan B Li Y Duan Y Co-dependence of genotype and dietary protein intake to affect expression on amino acid/peptide transporters in porcine skeletal muscle. Amino Acids (2016) 48:75–90. 10.1007/s00726-015-2066-2 26255284

[126] Xie C Wu X Li J Fan Z Long C Liu H Effects of the sequence of isocaloric meals with different protein contents on plasma biochemical indexes in pigs. PLoS One (2015) 10:e0125640. 10.1371/journal.pone.0125640 26295708 PMC4546430

[127] Patterson JK Lei XG Miller DD. The pig as an experimental model for elucidating the mechanisms governing dietary influence on mineral absorption. Exp Biol Med (2008) 233:651–64. 10.3181/0709-MR-262 18408137

[128] Sangild PT Siggers RH Schmidt M Elnif J Bjornvad CR Thymann T Diet- and colonization-dependent intestinal dysfunction predisposes to necrotizing enterocolitis in preterm pigs. Gastroenterology (2006) 130:1776–92. 10.1053/j.gastro.2006.02.026 16697741

[129] Blachier F M’Rabet-Touil H Posho L Morel M-T Bernard F Darcy-Vrillon B Polyamine metabolism in enterocytes isolated from newborn pigs. Biochim Biophys Acta (1992) 1175:21–6. 10.1016/0167-4889(92)90005-V 1482693

[130] M’Rabet-Touil H Blachier F Morel M-T Darcy-Vrillon B Duée P-H. Characterization and ontogenesis of nitric oxide synthase activity in pig enterocytes. FEBS Lett (1993) 331:243–7. 10.1016/0014-5793(93)80345-U 7690716

[131] Siggers RH Siggers J Thymann T Boye M Sangild PT. Nutritional modulation of the gut microbiota and immune system in preterm neonates susceptible to necrotizing enterocolitis. J Nutr Biochem (2011) 22:511–21. 10.1016/j.jnutbio.2010.08.002 21193301

[132] Blachier F Vaugelade P Robert V Kibangou B Canonne-Hergaux F Delpal S Comparative capacities of the pig colon and duodenum for luminal iron absorption. Can J Physiol Pharmacol (2007) 85:185–92. 10.1139/y07-007 17487259

[133] Stachowiak M Szczerbal I Switonski M Genetics of adiposity in large animal models for human obesity—studies on pigs and dogs. Progress in Molecular Biology and Translational Science. Elsevier (2016). p. 233–70. Available from: http://linkinghub.elsevier.com/retrieve/pii/S1877117316000028 10.1016/bs.pmbts.2016.01.001 27288831

[134] Koopmans SJ Schuurman T. Considerations on pig models for appetite, metabolic syndrome and obese type 2 diabetes: from food intake to metabolic disease. Eur J Pharmacol (2015) 759:231–9. 10.1016/j.ejphar.2015.03.044 25814261

[135] Heinritz SN Mosenthin R Weiss E. Use of pigs as a potential model for research into dietary modulation of the human gut microbiota. Nutr Res Rev (2013) 26:191–209. 10.1017/S0954422413000152 24134811

[136] Steinert R Feinle-Bisset C Geary N Beglinger C. Digestive physiology of the pig symposium: secretion of gastrointestinal hormones and eating control. J Anim Sci (2013) 91:1963–73. 10.2527/jas2012-6022 23307852

[137] Rist VTS Weiss E Eklund M Mosenthin R. Impact of dietary protein on microbiota composition and activity in the gastrointestinal tract of piglets in relation to gut health: a review. Animal (2013) 7:1067–78. 10.1017/S1751731113000062 23410993

[138] Klose V Bayer K Bruckbeck R Schatzmayr G Loibner A-P. In vitro antagonistic activities of animal intestinal strains against swine-associated pathogens. Vet Microbiol (2010) 144:515–21. 10.1016/j.vetmic.2010.02.025 20226602

[139] Etheridge R Seerley R Wyatt R. The effect of diet on performance, digestibility, blood composition and intestinal microflora of weaned pigs. J Anim Sci (1984) 58:1396–402. 10.2527/jas1984.5861396x 6746437

[140] Pieper R Villodre Tudela C Taciak M Bindelle J Pérez JF Zentek J. Health relevance of intestinal protein fermentation in young pigs. Anim Health Res Rev (2016) 17:137–47. 10.1017/S1466252316000141 27572670

[141] Zhou X-L Kong X-F Lian G-Q Blachier F Geng M-M Yin Y-L. Dietary supplementation with soybean oligosaccharides increases short-chain fatty acids but decreases protein-derived catabolites in the intestinal luminal content of weaned Huanjiang mini-piglets. Nutr Res (2014) 34:780–8. 10.1016/j.nutres.2014.08.008 25236424

[142] Liu Y Li F Kong X Tan B Li Y Duan Y Signaling pathways related to protein synthesis and amino acid concentration in pig skeletal muscles depend on the dietary protein level, genotype and developmental stages. PLoS One (2015) 10:e0138277. 10.1371/journal.pone.0138277 26394157 PMC4578863

